# Forming and implementing community advisory boards in low- and middle-income countries: a scoping review

**DOI:** 10.1186/s12910-019-0409-3

**Published:** 2019-10-17

**Authors:** Yang Zhao, Thomas Fitzpatrick, Bin Wan, Suzanne Day, Allison Mathews, Joseph D. Tucker

**Affiliations:** 1 University of North Carolina at Chapel Hill - Project China, No.2 Lujing Road, Guangzhou, 510095 China; 2 University of Washington School of Medicine, Seattle, USA; 3 Department of Maternal and Child Health, School of Public Health, Sun Yat-sen University, Guangzhou, China; 4 Institute for Global Health and Infectious Diseases, University of North Carolina at Chapel Hill, Chapel Hill, USA; 5 School of Medicine, University of North Carolina at Chapel Hill, Chapel Hill, USA; 6 Faculty of Infectious Diseases, London School of Hygiene and Tropical Medicine, London, UK

**Keywords:** Community advisory boards, Research ethics, Low- and middle-income countries, Scoping review

## Abstract

**Background:**

Community advisory boards (CABs) have expanded beyond high-income countries (HICs) and play an increasing role in low- and middle-income country (LMIC) research. Much research has examined CABs in HICs, but less is known about CABs in LMICs. The purposes of this scoping review are to examine the creation and implementation of CABs in LMICs, including identifying frequently reported challenges, and to discuss implications for research ethics.

**Methods:**

We searched five databases (PubMed, Embase, Global Health, Scopus, and Google Scholar) for publications describing or evaluating CABs in LMICs. Two researchers independently reviewed articles for inclusion. Data related to the following aspects of CABs were extracted from included publications: time, country, financial support, research focus, responsibilities, and challenges. Thematic analyses were used to summarize textual data describing challenges.

**Results:**

Our search yielded 2005 citations, 83 of which were deemed eligible for inclusion. Most studies (65) were published between 2010 and 2017. Upper-middle-income countries were more likely to have studies describing CABs, with South Africa (17), China (8), and Thailand (7) having the greatest numbers. The United States National Institutes of Health was the main source of financial support for CABs. Many CABs (53/88, 60%) focused on HIV research. Thirty-four studies reported how CABs influenced the informed consent process for clinical trials or other aspects of research ethics. CAB responsibilities were related to clinical trials, including reviewing study protocols, educating local communities about research activities, and promoting the ethical conduct of research. Challenges faced by CABs included the following: incomplete ethical regulations and guidance; limited knowledge of science among members of communities and CABs; unstable and unbalanced power relationships between researchers and local communities; poor CAB management, including lack of formal participation structures and absence of CAB leadership; competing demands for time that limited participation in CAB activities; and language barriers between research staff and community members. Several challenges reflected shortcomings within the research team.

**Conclusions:**

Our findings examine the formation and implementation of CABs in LMICs and identify several ethical challenges. These findings suggest the need for further ethics training among CAB members and researchers in LMICs.

## Background

Organizing research studies in low- and middle-income countries (LMICs) present a number of challenges, including difficulties in ensuring community consultation [1–3]. Many funders now require that clinical trials engage with participating communities [4–6]. Community engagement is the process of working collaboratively with relevant stakeholders to address health-related issues that concern them [7, 8]. Community engagement can empower communities by actively soliciting input from potential research subjects, involving them in decision making, and ensuring their perspectives, attitudes, and values are respected [9]. Moreover, community engagement can build community trust in research, facilitate participant enrollment, and assist in post-trial implementation [4, 6, 8, 10].

Community advisory boards (CABs) are a well-established form of community engagement to strengthen research ethics [8, 11, 12]. The United States (US) National Institutes of Health (NIH) officially mandated CAB inclusion in all HIV clinical trials in 1987 [13]. CABs are typically composed of diverse individuals selected to represent researched communities [14]. Through organizing activities such as community consultations and regular feedback meetings, CABs provide trial participants and potential participants with an opportunity to understand the research process and voice concerns [15]. CABs also advocate for trial participants and promote the ethical conduct of research [14, 15].

Over the past three decades, CABs have become a standard mechanism for community engagement in HIV clinical trials conducted in high-income countries (HICs) [14, 16–18]. With the growth of the global NIH HIV trials network [19–22], CABs are increasingly formed in LMICs [14, 21]. While much research has examined CABs in HICs [16, 22, 23], these findings may be less relevant in LMIC settings. A growing literature has described CABs in LMICs over the past decade [16, 17, 24–26]. CABs in LMICs operate in environments that are distinct from their HIC counterparts. A range of economic, cultural, social, and historical considerations may constrain CABs in LMICs. The purposes of this scoping review are to provide an overview of the published literature describing the formation, implementation, and frequently reported challenges of CABs in LMICs, and to discuss implications for research ethics.

## Methods

### Identifying the research question and relevant studies

This scoping review followed the methodological framework for conducting a scoping study proposed by Arksey and O’Malley [27]. We first defined the objectives of our scoping review as providing an overview of the published literature describing the formation and implementation of CABs in LMICs, including relevant challenges faced by CABs in LMICs, and discussing implications for research ethics. We then identified relevant studies through an online search. Five databases were searched (PubMed, Embase, Global Health, Scopus, and Google Scholar) for English-language entries examining CABs in LMICs published before 01 December 2017. Search terms included “community advisory*” OR “community advisory board*” OR “community advisory group*” OR “community steering committee*” OR “community constituency group*” OR “community collaborative board*” OR “community working group*”.

### Selecting studies for inclusion

**Fig. 1:**
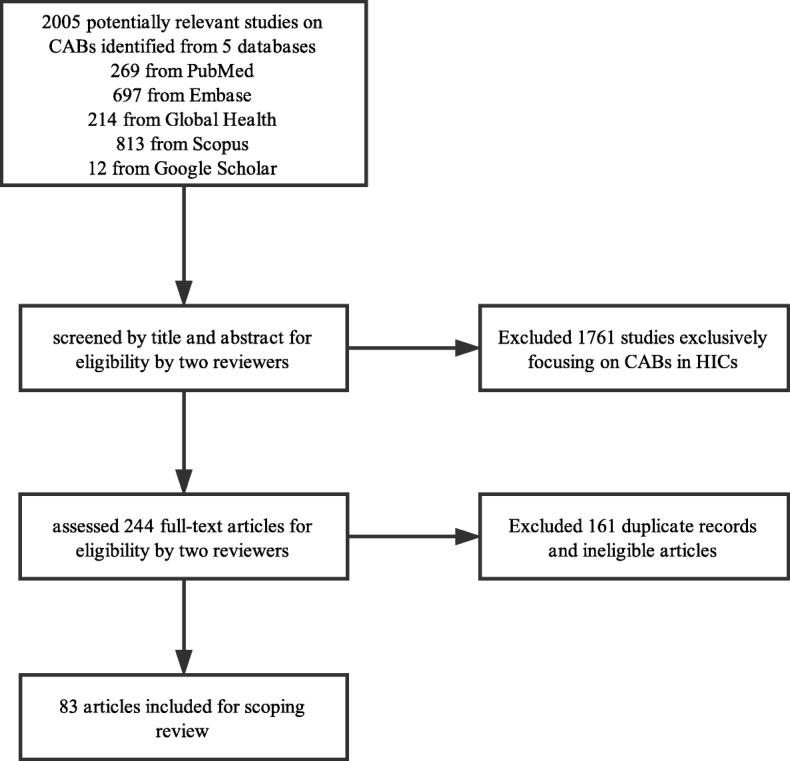
Selection of included studies

Two reviewers (YZ, BW) independently screened titles and abstracts for inclusion. Titles and abstracts focusing on CABs in HICs were excluded. Disagreement about inclusion or exclusion of abstracts was discussed and resolved by consensus between the same two reviewers. Full-text publications were then screened by two reviewers (YZ, BW) who ensured all articles included descriptions of the formation or implementation of a CAB, discussions of the challenges involved in operating a CAB, or explorations of the possibility of organizing a CAB in an LMIC. Studies only referring to a CAB without mentioning related characteristics or in-depth discussion were excluded. Included publications were peer-reviewed articles, book chapters, and detailed summaries of research presented at international conferences. Our scoping review primarily focused on the peer-reviewed full articles, but other publications, including abstracts, were also included to supplement findings and present a more comprehensive overview of CABs in LMICs. The process of study selection is outlined in Fig. 1.

**Fig. 2:**
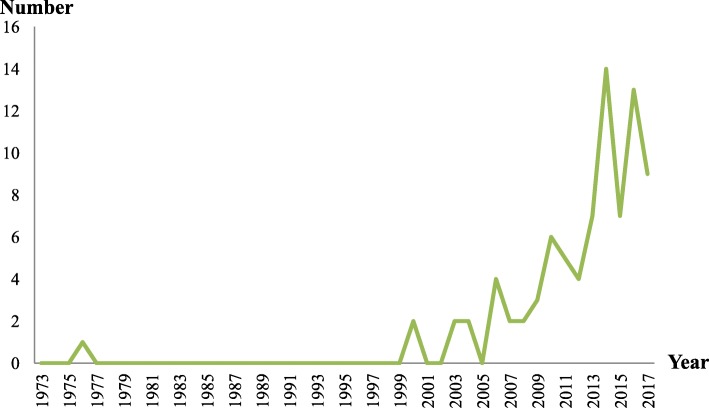
Number of included publications describing CABs in LMICs by year

### Charting the data

Data related to the following aspects of CABs were extracted from all publications selected for inclusion: year of publication, country or region in which the CAB was operated, membership, activities, funding sources, research focus, achievements, and challenges. Thematic analyses were conducted to summarize and categorize extracted data.

## Results

Our initial search identified 2005 potentially relevant publications. After screening titles, abstracts, and full-text manuscripts, 83 publications met selection criteria and were included in our analysis. Sixty-three were peer-reviewed articles, fifteen were international research conference abstracts, three were presentations from international research conferences, and two were book chapters. Two articles published in Chinese with online English abstracts were included as abstracts in our analyses. All abstracts and presentations included a year of publication and country or region, most described a research focus, and only a few presented specific challenges. Twenty publications specifically researched a CAB in an LMIC using a qualitative approach. The remaining 63 publications described the function of a CAB in the context of a larger research project.

Year of publication for included publications ranged from 1976 to 2017. Of the 83 included publications, 78% (65) were published after 2009, and 52% (43) were published within the last 4 years (Fig. 2). Only one study was published prior to 2000. The 1976 publication proposed establishing a CAB to improve the health of rural residents in Thailand [28].

The included publications described 88 different CABs across 26 LMICs and nine geographic regions in Africa, Asia, Oceania, South America, and Europe. The five LMICs with the most CABs were South Africa (17), China (8), Thailand (7), Zimbabwe (6), and India (5). CABs in Africa (*n* = 49, 56%) and Asia (*n* = 24, 27%) were disproportionately represented among 88 CABs described in the included publications. Only four CABs were based in South America. Nine studies did not focus on a specific country, but rather a broader geographic region or economic setting (e.g., West Africa, Pacific Islands). Additionally, CABs in LMICs were unevenly distributed across countries in terms of income level. CABs were disproportionately represented in upper-middle-income countries, particularly South Africa, China, and Thailand. In total, thirty-nine CABs in upper-middle-income countries were described, accounting for nearly half of all the CABs identified in this review. In comparison, only 20 and 19 CABs were in lower-middle-income and low-income countries, respectively.

We found 41 included publications provided details on CAB membership and 24 on the process of CAB membership selection. Members were selected through elections by community members [26], purposive sampling selections by the research team, community members, and sometimes stakeholders [13, 18, 23–25, 29–37], or mixed methods combining several of these strategies [16, 24]. A common theme in the descriptions of CAB formation was promoting diversity in CAB membership to ensure adequate representation from key groups. We identified gender, age, religion, class, ethnicity, and religious affiliation as the most commonly used selection factors used to promote diversity in CAB membership. Additionally, CABs also proactively included members from stakeholder groups specific to certain LMIC contexts, such as religious leaders [16, 18, 19, 35, 38–40], traditional healers [16, 18], individuals from different villages, cities, and countries [17, 18], media representatives [39], non-governmental organizations workers [31, 41], local politicians [42], patients and family members [19, 43–45], refugees [46], and groups at increased risk of HIV infection (e.g., sex workers, men who have sex with men, and miners) [16, 19, 41, 47].

CAB activities included training sessions, periodic meetings, focus group discussions, site visits, conference calls, and group emails. Ten articles described training sessions for CAB members, including technical medical expertise trainings, CAB and community leadership skills improvement sessions, protocol specific training, report writing guidance, and protection of research participants, which typically lasted several days and were uniformly conducted before CABs began operating in their advisory capacity [13, 18, 26, 33]. Most CAB members had no formal medical or public health training, and consequently organizing teams (e.g. research teams, staff from professional training institutions, and universities) tended to focus training on improving members’ medical knowledge as well as addressing research goals and ethics [13, 18, 33, 38, 45, 48, 49]. Once formed, most CABs held periodic meetings, with meeting frequently ranging from weekly to monthly to quarterly to yearly. Meetings typically focused on reporting and discussing progress in preparing, designing or implementing clinical trials (e.g. updates on study findings and current ethical challenges). Thirty-four studies reported how CABs influenced clinical trial informed consent or other aspects of research ethics. CAB activities also included educating communities about research activities and facilitating qualitative research related to ongoing clinical trials.

Most CABs in LMICs were supported through funding from the NIH or other research institutions based in HICs. Among the 34 CABs whose funding sources were reported, 41% (14) were part of projects that were entirely funded by the NIH. Seven CABs were part of projects that were funded by European institutions, including the Wellcome Trust (4), National Belgian Lottery Fund (1), and German Federal Ministry of Education and Research (1). Eleven CABs received financial support by being part of projects from both domestic and foreign sources. For example, a CAB in Tanzania was part of a project that was funded by the Ifakara Health Institute with support from both Switzerland and Tanzania, and a CAB in India received funding by being part of a project from the Indian Council of Medical Research and the NIH [37]. Only one CAB located in South Africa received funding exclusively from domestic sources.

**Fig. 3:**
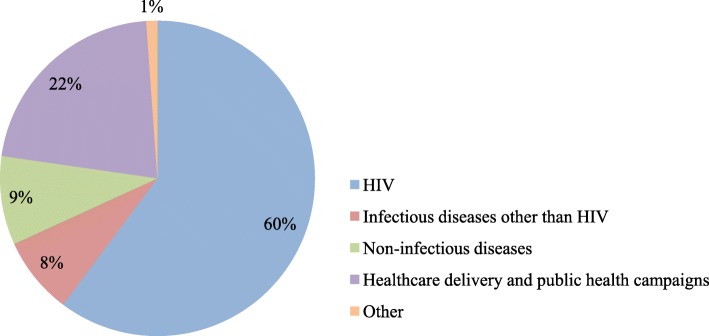
CAB research foci in LMICs

Most CABs (53 publications, 60%) in our sample advised on HIV research [50–60], vaginal microbicides [61, 62], and antiretroviral medication adherence [37]. Seven CABs advised on research related to other infectious diseases, including tuberculosis [17, 18] and malaria [38, 63]. Eight CABs focused on research for non-infectious diseases, including stroke, cancer, malnutrition, and mental illness [30, 35, 39, 64–69]. 19 CABs did not address a specific disease and instead focused on healthcare delivery and public health campaigns generally, including rural and primary healthcare services [46, 70–77]. Finally, one CAB advised on non-medical research, namely, earthquake preparedness [78]. CAB research foci are presented in Fig. 3.

Fourteen of the included publications provided detailed descriptions of CAB achievements. According to these descriptions, CABs in LMICs improved recruitment strategies, enhanced relationships between researchers and local communities, and addressed ethical concerns related to participant risk in collaboration with institutional review boards (IRBs). For example, the CAB in a South African schizophrenia genomics study helped individual research participants decide about data sharing [25]. Some researchers may lack specialized knowledge of specific locations and sub-cultures within a diverse region [64]. As part of the community, CABs were often presented as a ‘bridge,’ ‘belt,’ ‘link,’ or ‘intermediary’ that helped to solidify partnerships between researchers and community members [13, 16]. CABs were described as an efficient tool to ensure that CAB and community members understood the goals of clinical trials by providing detailed explanations and facilitating discussions [35, 79]. Informed consent forms were identified as complex, legalistic, and difficult to understand for vulnerable populations in LMIC settings. CABs addressed concerns with informed consent language [79]. CABs also contributed to study recruitment by refining recruitment strategies and protecting research participants and the wider community from research-related risk [25].

Challenges faced by CABs in LMICs were specifically addressed by 62% (39) of the 63 included articles and 35% (7) of the included abstracts and presentations. After performing thematic analysis of reported challenges, we categorized challenges facing CABs in LMICs into six main types: (1) incomplete ethical regulations and guidance, (2) limited knowledge of science, (3) competing demands, (4) unbalanced power relations, (5) poor CAB management, and (6) language barriers.

### Incomplete ethical regulations and guidance

Thirteen publications highlighted the absence of guidance on ethical regulations as having negatively impacted the work of CABs in LMICs [5, 16, 24, 31, 46, 79–86]. Incomplete ethical regulations may engender harm or result in exploitation of participating communities, particularly in LMICs where populations are often disadvantaged, marginalized, or under-resourced. Because of the possibilities of exploitation and unfair benefits in conducting research mentioned by the members of CABs in HIV vaccine trials in South Africa, CAB members and researchers in certain situations felt unprepared to address the tension between generating new knowledge and protecting participants from harm [24]. Essential components of the ethical conduct of clinical research were sometimes unclear to CAB members. For example, CAB members felt uncertain how to ethically enroll minors in sexual and reproductive health research studies, particularly those in early child marriages whose parents did not have parental custody and control [82]. Consequently, CABs may have been unable to adequately perform their function of protecting local participants in research [16, 82, 83]. Given larger power imbalances between CABs in LMICs and researchers from HICs, CAB members at these sites pointed out the need for research projects to leave behind a lasting benefit for their communities [16].

### Limited knowledge of science

Twenty-three publications highlighted knowledge gaps between researchers and members of communities and CABs [5, 13, 16, 17, 25, 26, 31–37, 40, 41, 44, 79–81, 84, 85, 87, 88]. It was noted that average members of the population in LMICs, including CAB members, were more likely to have lower levels of education compared to HICs [5, 35]. Consequently, authors expressed concerns about CAB members not fully comprehending the nature of the study and concepts being researched [13, 16, 17, 32, 40, 87, 88]. Since members of communities and CABs had different levels of education and social backgrounds compared to researchers, they may not have fully understood or appreciated researchers’ viewpoints, and researchers may have lacked the cross-cultural knowledge and skills necessary to make this knowledge accessible to local communities [13, 17, 25, 31, 87]. For instance, CAB members at one institution were at times unable to explain detailed research procedures to inform communities. This was not only due to the limited science knowledge of participating communities, but also related to the lack of cross-cultural understanding and skills on the part of researchers [33]. Inadequate consideration of community perspectives on the part of research teams has contributed to miscommunication in many research studies [31].

### Unstable and unbalanced power relations

Unstable and unbalanced power relations among CAB members and between CAB members and researchers were identified in 18 publications [5, 18, 24, 26, 31, 33, 35, 36, 38, 45, 57, 63, 64, 81, 87, 89–91]. CAB members were sometimes less likely to challenge the opinions of doctors and researchers because of internalized perceptions that it was inappropriate to question those in positions of authority [5]. Political tensions were specifically identified as impacting CAB operations in China [89], South Africa [24], and along the Thai-Myanmar border [5, 38, 63]. The diversity of religious, political, language, and ethnic groups along the Thai-Myanmar border raised questions as to what constituted a community and who may serve as a community representative. Moreover, due to incomplete CAB membership selection criteria [87], marginalized groups tended to be overlooked during CAB formation. For instance, the electoral model used to establish CABs at the HIV vaccine trial sites in South Africa was not purely democratic, as they elected one person to represent all the 50 families during an ad hoc meeting [24]. The selection of CAB representatives was not always democratic. One CAB was composed of individuals selected only by chiefs or health care workers [33]. The membership and participation of some women in a South African CAB were limited due to the impact gender-based violence on the selection process [29, 87]. Few CABs used an electoral model to select members, and many CABs lacked a formal structure for participation [5, 33]. These complex and politically sensitive issues were difficult for CAB organizers to resolve [45]. Efforts to implement CAB activities among target populations were impeded by social hierarchies due to age, gender, experience, and social status in several contexts [5, 31, 57, 91].

### Poor CAB management

Poor CAB management was discussed as a challenge in fourteen publications [5, 13, 17, 18, 24, 26, 29, 33, 34, 38, 81, 85, 87, 92]. Some researchers were inadequate in providing targeted and appropriate trainings for CAB members [17, 26, 34, 81, 85]. Due to poor training, some CAB members may not have developed the skills necessary to provide feedback to researchers and liaise with their peers. For example, some research teams acknowledged that in the beginning of their clinical trial they did not engage with the CABs in Malawi, invite them to meetings, or provide them with the necessary background information on the clinical trial [26]. Consequently, the broader community may not have appreciated the value of CAB engagement [13, 17]. Additionally, several CABs lacked leadership structures, which negatively impacted CAB functionality [13, 33]. Some CABs were also required to work on several research projects simultaneously, potentially affecting members’ ability to sufficiently focus on particular research issues [18, 38].

### Competing demands

Thirteen publications reported that CAB attendance was negatively affected by other competing demands [5, 13, 16, 17, 26, 32, 33, 35, 37, 41, 64, 81, 85]. Professional and social obligations, including employment, business dealings, and social activities [33], prevented members from consistently participating in CAB meetings and activities. The demands of full-time employment were specifically identified as being a challenge for CABs in four publications [13, 33, 35, 81]. For example, competing work demands limited the number of participants who voted at one CAB [33]. Limited economic resources and insufficient compensation for CAB participation were identified as a cause of CAB member dissatisfaction in several included publications [17, 33, 85]. For example, community leaders in five studies reported difficulty convincing CAB members to regularly attend CAB meetings because of insufficient participation incentives [16, 26, 37, 41, 64]. However, there was also disagreement as to whether participation in CABs should be driven by financial incentives or a sense of volunteerism that was primarily motivated by altruism [13].

### Language barriers

Language barriers were identified as a challenge in eleven publications [5, 35, 36, 38–40, 85, 91, 93–95]. CAB members sometimes had low literacy levels and could not sign their name or read documents related to research projects [5, 40]. Multiple languages and dialects were spoken across or within several of the communities in which CABs were based, creating challenges for communication between researchers and CAB members [35, 38, 39, 91]. English proficiency was uncommon among CAB members in LMICs [35, 36, 85], and in certain situations the number of translators was insufficient [93], which further hindered communication. Researchers also tended to have limited abilities in speaking local languages or dialects, although few articles discussed this limitation in detail. One CAB along the Thai-Myanmar had twelve of the fourteen founding members unable to speak proficient English, and researchers could not understand the diverse local languages and dialects, which made communication between researchers and the CAB challenging [5],

## Discussion

This scoping review examines the formation, operation, and challenges of CABs in LMICs and discusses implications for research ethics. Many CABs have focused on HIV clinical trials in upper-middle income countries. Some studies described how CABs influenced the informed consent process and helped address ethical concerns in clinical trials. Several challenges in implementing CABs in LMICs were identified, particularly challenges related to ethics guidance and regulations.

Most of the CABs in LMICs described in the included publications focused on HIV. This may be due to the growth of the global NIH HIV trials network in LMICs and HIV trials being more likely to publish on CAB-related experiences. In contrast, CABs in HICs have advised on more diverse clinical trials [96–104], ranging from cardiovascular disease to mental health. This difference in breadth of research foci may be due to the fact that the majority of NIH-funded trials in LMICs have investigated HIV. As the number of chronic disease trials increase in LMICs [105, 106], CABs may be able to expand as well. Some CABs originally focused on HIV have already transitioned to include chronic diseases [25, 30, 35, 39, 64, 107].

We found that most CABs described in the existing literature were based in upper-middle-income countries. Only 19 of the CABs included in this review were based in low-income countries. This finding is consistent with prior reviews which found that few studies have investigated community engagement in low-income countries [8, 108, 109]. Because clinical trials are often conducted in low-income countries [110–112], future investigations of CABs should focus on the accomplishments and challenges specific to this context.

Limited knowledge of science among members of communities and CABs was a common challenge noted among CABs in LMICs. Most included publications attributed this challenge to the different levels of education and social backgrounds and experience between researchers and members of communities and CABs. Eighteen publications mentioned that community members had better knowledge of local cultural and social contexts compared to researchers [5, 12, 13, 24, 25, 28, 29, 31, 36, 39, 43, 48, 50, 57, 62–64, 84]. Eleven publications also discussed community perceptions of the limitations of researcher-community connections in LMICs related to knowledge gaps and failure to provide enough training in science [13, 16, 17, 24, 31, 33, 35, 40, 85, 88, 95]. It is worth noting that researchers may also have lacked the skills necessary to communicate scientific knowledge in understandable and meaningful ways to participating communities. CAB training helped to build capacity and knowledge among members and researchers [113, 114]. This also suggests the need for further CAB member and researcher training [16]. Limited knowledge of science was closely connected with language barriers, another challenges that exposed the limitations on the parts of both CAB members and researchers.

Our data suggest that CAB members sometimes had insufficient ethics guidance. Some researchers were inadequate in providing targeted and appropriate trainings for CAB members [17, 26, 34, 81, 85], and only seven publications mentioned CAB members had received training on ethics [13, 18, 33, 38, 45, 48, 49]. Thirteen publications highlighted the absence of guidance on ethical regulations of CABs in LMICs and suggested the importance of ethics training [5, 16, 24, 31, 46, 79–86]. Several ethics guidance documents related to HIV research have been developed [115–117]. However, our review suggests these documents have not been widely incorporated into standard CAB operations in LMICs. Future research in LMICs should consider incorporating pre-existing ethics documents, including ethics guidelines, statements of shared principles, and other contextualized ethics documents, into CAB member training so that CABs are better prepared to address ethical concerns.

Our study has several limitations. First, we identified challenges facing CABs in LMICs based on published literature, largely reflecting the perspective of research teams. There are fewer perspectives from communities themselves in the literature and our review is less able to capture these important perspectives. Second, our search was limited to English language publications. English is not the primary language in many LMICs, and we may have missed studies in local languages. In addition, the failure to search through Spanish and French language publications may have biased our sample. There may additional relevant publications written in local languages highlighting the challenges we did not identify in this review. Third, we included all types of publications, including peer-reviewed articles, international research conference abstracts and presentations. While this review primarily based its analysis on data extracted from peer-reviewed articles, we also included abstracts and presentations to reinforce and complement our findings. The different levels of information richness across publications may mean that some CAB characteristics were only reported by a subset of studies. Finally, this was not a systematic review. Inferences about CABs in other settings should be made with caution.

Our findings have implications for LMIC research ethics. First, CABs allow an opportunity to consider research ethics prior to formal IRB review [118]. CABs engage diverse community members and may facilitate deliberations about ethical concerns from the community which can improve the quality of research and pre-empt issues that may later be identified during formal IRB review [119]. Research ethics goes beyond IRB approval alone and CABs can help to broaden discussions about research ethics in communities [120]. Second, only some studies reported the extent to which CABs advising a clinical trial influenced informed consent and research ethics. This suggests the need for further research about how CAB activities impact, or fail to impact, the conduct of clinical trials. Third, our data suggest that capacity building is needed among both CABs in LMICs and researchers. Training for CAB members to clarify ethics regulations and theory may be useful. Researchers may also require additional training to better appreciate the importance of community engagement, improve communication skills, and potentially learn local languages or dialects used in participating communities. Finally, challenges arising from the local contexts were frequently reported in our included articles. Issues specific to LMIC contexts, such as the high prevalence of child marriage, should be fully understood and taken into consideration when CABs and researchers work together to address ethical challenges.

## Conclusions

CABs may be a useful tool for increasing community engagement and the ethical conduct of research in LMICs. This scoping review describes the implementation and operations of CABs in LMICs, including challenges faced by these organizations, and discusses the implications for research ethics. We found several shortcomings and opportunities for increasing CAB understanding of research ethics. These findings can help inform training and related activities to enhance CABs in LMICs.

## Data Availability

The original dataset is owned by the first author. Researchers interested in using these data can contact YZ with specific research questions and a proposal.

## Abbreviations

AIDS: Acquired Immune Deficiency Syndrome
CABs: Community Advisory Boards
HICs: High-income Countries
HIV: Human Immunodeficiency Virus
LMICs: Low- and Middle-income Countries
NIH: National Institutes of Health
US: United States
